# Rapid Generation of Barley Mutant Lines With High Nitrogen Uptake Efficiency by Microspore Mutagenesis and Field Screening

**DOI:** 10.3389/fpls.2018.00450

**Published:** 2018-04-06

**Authors:** Runhong Gao, Guimei Guo, Chunyan Fang, Saihua Huang, Jianmin Chen, Ruiju Lu, Jianhua Huang, Xiaorong Fan, Chenghong Liu

**Affiliations:** ^1^Biotechnology Research Institute, Shanghai Academy of Agricultural Sciences, Shanghai, China; ^2^Shanghai Key Laboratory of Agricultural Genetics and Breeding, Shanghai, China; ^3^College of Bioscience and Biotechnology, Co-innovation Center for Modern Production Technology of Grain Crops, Yangzhou University, Yangzhou, China; ^4^State Key Laboratory of Crop Genetics and Germplasm Enhancement, Ministry of Agriculture, Nanjing Agricultural University, Nanjing, China

**Keywords:** microspores, mutation, nitrogen use efficiency, nitrogen uptake efficiency, nitrogen utilization efficiency, mutant screening, barley (*Hordeum vulgare* L.)

## Abstract

*In vitro* mutagenesis via isolated microspore culture provides an efficient way to produce numerous double haploid (DH) lines with mutation introduction and homozygosity stabilization, which can be used for screening directly. In this study, 356 DH lines were produced from the malt barley (*Hordeum vulgare* L.) cultivar Hua-30 via microspore mutagenic treatment with ethyl methane sulfonate or pingyangmycin during *in vitro* culture. The lines were subjected to field screening under high nitrogen (HN) and low nitrogen (LN) conditions, and the number of productive tillers was used as the main screening index. Five mutant lines (A1-28, A1-84, A1-226, A16-11, and A9-29) with high numbers of productive tillers were obtained over three consecutive years of screening. In the fifth year, components related to N-use efficiency (NUE), including N accumulation, utilization, and translocation, were characterized for these lines based on N uptake efficiency (NUpE), N utilization efficiency (NUtE), and N translocation efficiency (NTE). The results show that the NUpE of four mutant lines (A1-84, A1-226, A9-29, and A16-11) improved significantly under HN, whereas that of two lines (A1-84 and A9-29) improved under LN. As a result, their NUE improved greatly. No improvement in NUtE was observed in any of the five mutant lines. A1-84 and A9-29 were selected as an enhanced genotype in N uptake, and A1-28 showed improved NTE at the grain-filling stage. Our results imply that high-NUpE mutants can be produced through microspore mutagenesis and field screening, and that improvement of NUE in barley depends on enhancement of N uptake.

## Introduction

Nitrogen (N) is an essential macronutrient for crop growth and development, which is usually required to achieve maximum yields in modern agriculture (Wu et al., 2011). Over the past four decades, the global production of agricultural food has doubled with a sevenfold increase input of N fertilizers (Hirel et al., 2007). However, only 30–40% of applied N fertilizers are absorbed by plants during crop production (Raun and Johnson, 1999; Kindu et al., 2014). Excessive use of N fertilizers has caused severe environmental problems, resulting in enrichment of the atmosphere, soil, and agricultural water runoff with reactive N (Ju et al., 2009). As China is the largest producer and consumer of N fertilizers, and the management of N pollution including N-related greenhouse gas emissions was more urgent and essential to global efforts (Zhang et al., 2013). Improved crop varieties with high N-use efficiency (NUE) are considered the best economic strategy for agricultural sustainability (Quan et al., 2016). Previous studies have shown that genetic improvement of NUE in crops is feasible (Ding et al., 2005; Namai et al., 2009; Quan et al., 2016). However, the narrow genetic base of barley has limited genetic improvement of NUE.

In recent years, the haploid system has been used to induce genetic variation through mutagenesis (Jambhulkar, 2007). Microspores are immature precursors to pollen grains, which retain the potential to form haploid embryos under specific conditions that eventually develop into whole plants. The unique developmental pathway is referred to as microspore embryogenesis (Touraev et al., 1997), which is widely exploited for plant breeding and served as an efficient tool to produce DH plants (Soriano et al., 2013). Microspore is an ideal target for mutation induction and selection. it provides several advantages over the traditional seed mutagenesis method. Microspore is far more sensitive to mutagenic treatments than seeds, and it is easy to handle in a tube or dish with large number of microspores at lower dose. The key factor is the ability of fixing mutation in a complete homozygous state via doubled haploidy (Szarejko and Forster, 2007), which facilitates the screening of both recessive and dominant mutants as early as M1 generation, avoiding chimerism and shortening the breeding time (Germanà, 2011). As an attractive approach, microspore mutagenesis has been successfully applied in several cruciferous plants (Barro et al., 2001; Beaith et al., 2005; Ferrie et al., 2008; Liu et al., 2010; Huang et al., 2016). The physical treatments often applied to microspores were gamma rays and UV radiation, and the most common chemical mutagen was EMS (Szarejko and Forster, 2007). In addition, the antibiotic PYM was reported as a safe and efficient mutagen in crop development (Zeng et al., 2010; Zhao et al., 2013; Sui et al., 2015). In this study, the chemical mutagens EMS and PYM were selected to treat the isolated microspores prior to *in vitro* culture.

However, the application of microspore mutagenesis has been limited by the efficiency of isolated microspore culture system. In our previous work, an efficient protocol of isolated microspore culture has been established for an elite barley variety Hua-30, which was cultivated in the area of Yangtze River Delta of China for malt barley production (Lu et al., 2008, 2016). In this study, a M1 generation with homozygous DH lines was rapidly obtained from barley cv. Hua-30 by microspore mutagenesis and *in vitro* screening culture in low N medium, and the lines were subjected to further identification in fields under HN and LN conditions. The N absorption, translocation, and utilization of Hua-30 and of five selected mutant lines were studied and two outstanding mutant lines with improved NUpE were identified under both HN and LN conditions. Our results reveal that microspore mutagenesis combined with field screening is an efficient approach to generate homozygous mutant lines for genetic improvement of NUE.

## Materials and Methods

### Plant Materials and Growth Condition

The barley cultivar Hua-30 was used as the WT for all experiments. Isolated microspores were treated with EMS or PYM and then cultured in LN medium to generate plants. Homozygous DH lines were obtained by natural chromosome doubling and reproduction in Kunming (Yunnan Province) between April 2010 and October 2011. The seeds of the M0 generation were harvested from each single plant separately, and the derived DH lines (M1 generation) were screened under two levels of N in a field (Qingpu District, Shanghai) from 2011 to 2015. The barley was cultivated in crop rotation with rice as a preceding crop. The soil type was clayey soil with the following properties: pH 6.3, 2.23 g kg^-1^ total N, 2.29% organic matter, 3.02 mg kg^-1^ available P and 70.42 mg kg^-1^ available K. Data were collected in 2014–2015.

### Isolated Microspore Culture

The isolated microspore culture procedures were conducted as described by Lu et al. (2008), except for the microspore mutagenesis. Briefly, spikes were collected and pretreated at 4°C for 15 days. The isolated microspores were treated with chemical mutagens EMS (8.0, 24.0, and 40.0 mM) and PYM (2.0 and 3.0 μM) independently in extraction buffer at 25°C for 48 h in dark. The buffer was comprised of 330 mM mannitol, 10 mM calcium chloride (CaCl_2_), and 5 mM MES hydrate (Sigma–Aldrich, United States). The tested concentration range of chemical mutagens was determined according to the previous reports (Barro et al., 2001; Zeng et al., 2010). The treated microspores were washed with the induction medium and adjusted to a density of 5.0 × 10^5^ microspores⋅mL^-1^ for further culture. The sample was divided into four Petri dishes. The yield of embryogenic callus was defined as the wet weight of the callus (aspirated all medium out) formed in each Petri dish after 21 days of culture. The yield of green plants was defined as the number of green plants produced by 100 mg callus. N6 medium was used as the basic medium with some modifications for callus induction and supplemented with 2.3 μM KT, 4.5 μM 2, 4-D and 260 mM maltose. The differentiation medium was based on 9.5 mM agar-solidified MS and supplemented with 2.2 μM 6-BA, 7.0 μM KT, 0.3 μM NAA, and 88 mM maltose. The LN medium was 1/2 inorganic N and 1/4 organic N of the normal induced medium. The extraction buffer and induction medium were sterilized by filtration and the differentiation medium was sterilized in an autoclave (0.11 Mpa, 121°C for 15 min).

### Field Screening

The M1 generation was grown in a field (Qingpu District, Shanghai) to screen for mutants with high NUE. In the first round, two N levels (defined as N-fertilized and N-unfertilized) were used in the field experiments in 2011–2012 and 2012–2013. The level of N-fertilized was 160 kg hm^-2^ of pure N, which was divided into two parts: base fertilizer (compound fertilizer, 15% N concentration) and topdressing (urea, 46.7% N concentration). The base fertilizer accounted for 28% of the total N fertilizer, and was applied before sowing; the topdressing accounted for 72% of the total fertilizer and was applied at the tillering stage in twice. There was no N fertilizer input for the treatment of N-unfertilized level. In the second round, two N levels (HN and LN) were used in the field tests over 2 years (2013–2014 and 2014–2015). High N (HN) comprised 160 kg hm^-2^ of pure N, composed of base fertilizer (45 kg hm^-2^) and topdressing (115 kg hm^-2^), and low N (LN) comprised 45 kg hm^-2^ of pure N, with only base fertilizer applied. According to Baethgen et al. (1995), approximately 30 kg hm^-2^ of pure N was necessary for barley to ensure adequate crop establishment and initial tiller development. In order to get sufficient grain production for investigation we set 45 kg hm^-2^ of pure N as LN treatment. For the HN, we set 160 kg hm^-2^ of pure N, which was higher than the highest level of N fertilizers (120 kg hm^-2^ of pure N) reported by Baethgen et al. (1995), and it did not cause lodging to affect the yield in our field experiments. The fields with different N regime were separated by guard rows and ridges. The same N level was used within one field. The field was divided into small zones in the same size using strings to receive N fertilizers evenly during the application of base fertilizer and topdressing. For each treatment, four to five replicate blocks in a randomized block design were used. Plants were grown in 2.5-m-long rows, and there was a 16-cm gap between rows. In the first year (2011–2012), plant height, PTNs per plant, and grain yield per plant were investigated at harvest. In the following 2 years, only plant height and PTNs per plant were investigated at harvest. In the last year, the plant height, PTNs per plant, spike length, grain number per spike, seed setting rate, and grain yield per plant were investigated at harvest.

### Biomass, Total N Measurement, and Calculation of NUE

Fresh Hua-30 plants and five DH line plants were harvested with four replicates at 10:00 AM under two N levels. Each replicate had two plants and each plant had five tillers. Then, the ten tillers were divided into five parts including spikes, flag leaves, second leaves, other leaves, stems, and sheaths. All of the tissues were heated to 105°C for 30 min and dried at 75°C for 3 days. The dried plant tissue was ground and digested for total N determination using the Kjeldahl method (Li et al., 2008). A 5-mL aliquot from a total of 100-mL per digested sample was analyzed using a continuous-flow autoanalyzer (FlowSys, Systea, Anagni, Italy). The biomass values of plant part were calculated as dry weights per tiller multiplied by the number of tillers per plant. The biomass value of the whole plant above ground was the sum of the biomass values of all of the plant parts. Total N was estimated as the sum of the N contents of all of the plant parts. These indicators were investigated, including the DMA, DMM, SDMA, SDMM, total N accumulation at anthesis (TNAA), total N accumulation at maturity (TNAM), spike N accumulation at anthesis (SNAA), and spike N accumulation at maturity (SNAM) under the two N conditions. The NUE was calculated by the method of Moll et al. (1982), where NUE (g/g) is defined as the grain production per unit of supplied N, N-uptake efficiency (NUpE, g/g) was calculated as the total amount of N in the above-ground plant at harvest per unit of supplied N, and N-utilization efficiency (NUtE, g/g) was calculated as the grain yield per total amount of N in the aboveground plant at harvest. The dry matter and N translocation and translocation efficiency were calculated as in Chen et al. (2016) with minor modifications. Dry matter translocation (DMT, g/plant) was calculated as (dry matter at anthesis – spike dry matter at anthesis) – (dry matter at maturity – spike dry matter at maturity); DMT efficiency (DMTE, %) was calculated as ((DMT/(dry matter at anthesis – spike dry matter at anthesis)) × 100%; N translocation (NT, mg/plant) was calculated as (total N accumulation at anthesis – spike N accumulation at anthesis) – (total N accumulation at maturity – spike N accumulation at maturity); N translocation efficiency (NTE, %) was calculated as (NT/(total N accumulation at anthesis – spike N accumulation at anthesis)) × 100%.

### Statistical Analysis

All of the data were analyzed by one-way analysis of variance and LSD tests using SPSS software version 21. Different letters after the mean values indicate statistically significant differences between the Hua-30 and mutant lines at *P* < 0.05.

## Results

### Embryogenic Callus Induction and Green Plant Regeneration From Microspore Mutagenesis by EMS and PYM

To produce a large population of mutagenized lines from barley cv. Hua-30, isolated microspores of Hua-30 were treated with the two types of chemical mutagens (EMS and PYM) at low concentrations. Embryogenic callus was successfully induced from the treated microspores in a LN medium (Supplementary Figure S1). Eventually, regenerated plants were obtained from each treatment (**Table 1**). In total, 356 plants survived and set seeds after being transferred to the field. With the increase of mutagen concentration, the yield of embryogenic callus had a slight decrease. In order to obtain sufficient plants for screening, we did not test other concentrations. It should have the room to be improved in future. Comparing the effects of the two chemical mutagens on the isolated microspore culture, it was observed that higher yields of both embryogenic callus and green plants were achieved using EMS treatment. The results show that PYM was more detrimental to microspore embryogenesis than was the use of EMS. The possible reason may be related to the strong mutagenic effect of PYM on the alteration of chromosomal fragments, while EMS typically produces point mutations.

**Table 1:** Yield of embryogenic callus and green plants regenerated from Hua-30 via microspore mutagenesis.

Mutagen	Concentration	Yield of embryogenic callus (mg^⋅^dish^-1^)	Yield of green plants (plant^⋅^100 mg callus^-1^)	Number of seeded plants	Line code
EMS (mM)	8.0	95.7	59.6	22	A10
	24.0	78.9	138.2	249	A1
	40.0	72.5	182.5	58	A9
PYM (μM)	2.0	56.9	14.1	26	A16
	3.0	53.4	17.4	1	A18

*The yield of embryogenic callus was defined as the wet weight of the embryogenic callus (aspirated all medium out) formed in each Petri dish after 21 days of culture. The yield of green plants was defined as the number of green plants produced per 100 mg embryogenic callus. EMS, ethyl methane sulfonate; PYM, pingyangmycin*.

### Screening Mutant Lines Under Different N Conditions in Field Experiments

In the first round of screening, in total 229 mutagenized lines were investigated in a field experiment between 2011 and 2012. The results show a wide range of variation among these lines (Supplementary Table S1). In this population, three important agronomic traits (plant height, PTNs, and grain yield per plant) varied widely among lines, and the calculated coefficients of variation (CVs) ranged from 6.06 to 38.25%. Comparing the two levels of N-fertilizer, the CVs of the three agronomic traits among lines were slightly larger for the N-unfertilized treatment than for the N-fertilized treatment. Comparing the three agronomic traits, the CV of plant height (<10%) was smaller than the values of the other two traits, regardless of the level of N-fertilizer, which implies that plant height is relatively stable in response to N. The CV of PTN, and particularly that of grain yield per plant, showed larger variation, indicating that these two traits have strong selection potential in a population. Among the 229 lines tested, it was observed that the distribution of all three agronomic trait values did not conform to a normal distribution under either the N-fertilized or N-unfertilized condition, and values diverged more heavily under the N-unfertilized condition (Supplementary Figure S2). To determine a key index for screening mutant lines with improved NUE, three agronomic traits related to yield were selected for correlation analysis. The correlation coefficients of PTN and grain yield per plant were 0.83 and 0.85, in the condition of N-fertilized and N-unfertilized, which were much higher values than those of plant height and PTN (0.36 and 0.6) and the correlation coefficients of plant height and grain yield per plant (0.43 and 0.63). Therefore, PTN was selected as the main screening index in the field experiments in 2012–2015. In the experiment of 2013–2014, only three lines grew in HN field due to limited space available at the time of seeding, and the raining season during sowing may influence the performance of tested lines. The PTN of 16 mutagenic lines were significant higher than that of WT. The results of the 4-year experiment were shown in Supplementary Table S2. PTNs varied widely, not only among the screened lines but also among the experiments in different years. Combined with their excellent field performance, five lines (A1-28, A1-84, A1-226, A9-29, and A16-11) with PTNs that were significantly higher than the WT (Hua-30), at least in one year of field experiments, were selected for further analysis.

### Comprehensive Evaluation of the Agronomic Traits of Five Mutant Lines

The five mutant lines and the WT under HN and LN in the field experiment in 2014–2015 were subjected to a comprehensive evaluation of agronomic traits (**Table 2**). The plant heights of the five lines showed no significant differences compared to the WT, except for that of A16-11, which was significantly shorter than the WT under HN. In addition, A16-11 was significantly shorter than the other three lines (A1-84, A1-226, and A9-29) under HN and was shorter than A9-29 under LN. The PTN per plant of A16-11 was significantly higher than that of the WT, but spike length and grain number per spike decreased significantly under HN. Seed-setting rates were significantly higher in the mutant lines than in the WT under HN, and those of three of the five lines (A1-226, A9-29, and A16-11) were significantly higher than the WT under LN. The grain yields per plant of A1-84 and A1-226 were significantly higher than that of the WT under HN, and only that of A1-84 was significantly higher than that of the WT under LN. Although A1-226 had poor PTN values, it had the longest spikes and largest grain numbers per spike under HN, which were the main reasons for it achieving the highest grain yield. Under LN, although A9-29 had the longest spike, A1-226 had the largest grain numbers per spike and A16-11 had the highest PTN values. A1-84 achieved the highest grain yield per plant, which was significantly higher than the WT. This implies that improvement of one of these yield-related traits was not sufficient to improve grain yield per plant under LN. A1-226 had longer spikes, higher grain numbers per spike, and a higher seed-setting rate than the WT under both high and low N conditions, which is noteworthy for further studies of NUE genetic improvement.

**Table 2:** Comparison of agronomic traits between Hua-30 and mutant lines (2014-2015).

Nitrogen regime	Line	Plant height (cm)	Productive tiller number	Spike length (cm)	Grain number per spike	Seed setting rate (%)	Grain yield (g/plant)
HN	Hua-30	68.45ab	12.4b	7.63c	33.8b	85.74c	14.16c
	A1-28	64.71bc	12.9b	6.63d	27.7c	97.32a	14.45c
	A1-84	68.34ab	13.6ab	7.70c	34.0b	94.12b	18.34ab
	A1-226	71.98a	13.0b	9.17a	36.8a	98.45a	20.67a
	A9-29	69.83a	12.2b	8.63b	34.8b	98.79a	15.51bc
	A16-11	61.63c	16.5a	6.04e	24.3d	97.31a	14.53c
LN	Hua-30	56.20ab	6.8ab	6.73b	29.3b	92.33b	6.59b
	A1-28	56.54ab	8.3a	6.19c	26.0c	93.84ab	7.42ab
	A1-84	55.96ab	8.2a	7.13b	31.4ab	90.84b	8.39a
	A1-226	58.77ab	6.0b	7.73a	33.1a	96.82a	6.64b
	A9-29	62.17a	6.8ab	7.92a	32.5a	96.91a	7.54ab
	A16-11	53.11b	8.5a	5.75c	26.1c	96.80a	7.44ab

*HN indicates the treatment with 160 kg hm^-*2*^ of pure N input, and LN indicates the treatment with 45 kg hm^-*2*^ of pure N input. Different letters within the same column indicate significant differences at *P* < 0.05 by least significant difference (LSD) test. All data are means of four replicates*.

### Dry Matter and N Accumulation of the Five Mutant Lines

To analyze N allocation at the stages of anthesis and maturity, which are important for grain yields, the dry matter and N concentrations of the five mutant lines and the WT were quantified under LN and HN in the field in 2014–2015 (**Table 3**). Comparing the five mutant lines to the WT, the spikes and whole plants aboveground showed similar trends regarding differences in dry matter and N accumulation at the two stages. Under HN, four of the five lines (A1-84, A1-226, A9-29, and A16-11) produced significantly more dry matter and had higher N accumulation in spikes and in the whole plant aboveground at maturity. The values of DMM, SDMM, TNAM, and SNAM increased by approximately 15–36%, 16–41%, 30–49%, and 40–53%, respectively, compared to the WT. Under LN, four of the five lines (A1-28, A1-84, A9-29, and A16-11) produced significantly more dry matter and had higher N accumulation in spikes and in the whole plant aboveground at anthesis. The values of DMA, SDMA, TNAA, and SNAA increased by approximately 24–41%, 55–62%, 35–185%, and 80–223%, respectively, compared to the WT. The trend in the difference shifted from maturity to anthesis when the N regime changed from HN to LN. In addition to these differences, A1-226 had significantly higher TNAA than the WT under HN. Under LN, the SDMM, TNAM, and SNAM values of A1-84 were significantly higher than those of the WT. The DMM, SDMM, and TNAM values of A9-29 were significantly higher than those of the WT. In general, A1-226 and A9-29 produced more dry matter and accumulated more N than the other lines under HN, either in the whole plant aboveground or in spikes. Under LN, A1-84 and A9-29 produced more dry matter and accumulated more N than the other lines, either in the whole plant aboveground or in spikes.

**Table 3:** Comparison of dry matter accumulation and N content between Hua-30 and mutant lines (2014-2015).

Nitrogen regime	Line	DMA (g/plant)	DMM (g/plant)	SDMA (g/plant)	SDMM (g/plant)	TNAA (mg/plant)	TNAM (mg/plant)	SNAA (mg/plant)	SNAM (mg/plant)
HN	Hua-30	30.74ab	34.49c	6.55a	17.87d	329.13b	340.31c	106.53a	262.54c
	A1-28	27.06b	31.32c	6.53a	16.81d	321.20b	390.13bc	107.63a	310.77bc
	A1-84	32.33a	40.31b	6.91a	20.69c	361.52ab	443.49ab	117.77a	367.22ab
	A1-226	32.97a	46.81a	7.20a	25.12a	410.80a	507.90a	129.64a	399.63a
	A9-29	35.13a	43.28ab	7.60a	23.62ab	380.79ab	500.10a	128.09a	401.30a
	A16-11	31.87ab	39.81b	7.09a	21.98bc	388.85ab	460.42a	119.23a	369.32a
LN	Hua-30	12.87b	16.27bc	2.78b	8.57c	100.05d	127.14c	38.97c	102.90bc
	A1-28	15.93a	16.96abc	4.50a	9.36bc	145.90b	150.21bc	74.54b	127.35bc
	A1-84	18.16a	18.22ab	4.34a	10.05ab	285.09a	315.07a	125.86a	212.92a
	A1-226	13.48b	15.40c	3.12b	8.68c	117.32cd	124.91c	49.32c	99.69c
	A9-29	17.70a	18.75a	4.30a	10.75a	155.39b	171.44b	72.98b	139.37b
	A16-11	16.38a	16.74abc	4.47a	9.40bc	135.42bc	140.29c	70.31b	115.56bc

*DMA, dry matter at anthesis; DMM, dry matter at maturity; SDMA, spike dry matter at anthesis; SDMM, spike dry matter at maturity; SNAA, spike nitrogen accumulation at anthesis; TNAM, total nitrogen accumulation at maturity. HN indicates the treatment with 160 kg hm^-*2*^ of pure N input, and LN indicates the treatment with 45 kg hm^-*2*^ of pure N input. Different letters within the same column indicate significant differences at *P* < 0.05 by least significant difference (LSD) test. All data were means of four replicates*.

### N Allocation at Anthesis and Maturity of the Five Mutant Lines

**FIGURE 1:**
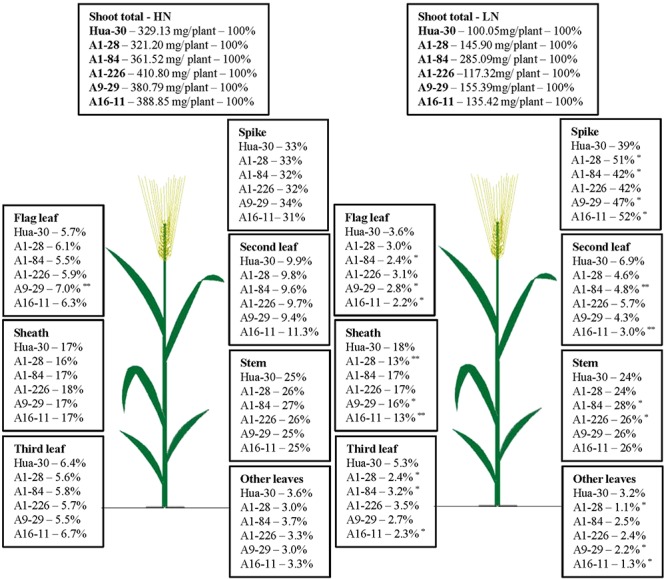
Nitrogen content percentage (%) of each tissue in Hua-30 and mutant lines under HN or LN at anthesis. ^∗^ and ^∗∗^ indicate significant differences at the 0.05 level and 0.01 level, respectively, compared to Hua-30 by the *t*-test. HN indicates the treatment with 160 kg hm^-2^ of pure N input, and LN indicates the treatment with 45 kg hm^-2^ of pure N input. All of the data are means of four replicates.

**FIGURE 2:**
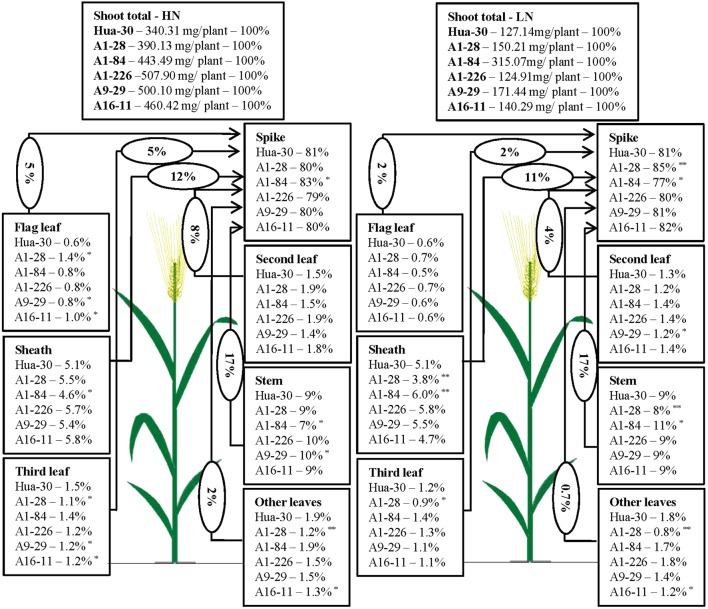
Nitrogen content percentage (%) of each tissue in Hua-30 and mutant lines under HN or LN at maturity. Arrow indicates the direction of different sources (leaves, stem, and sheath) to sink (spike). The percentage in the ellipse is the percentage of nitrogen transport from the different sources to the sink after anthesis. ^∗^ and ^∗∗^ indicate significant differences at the 0.05 level and 0.01 level, respectively, compared to Hua-30 by the *t*-test. HN indicates the treatment with 160 kg hm^-2^ of pure N input, and LN indicates the treatment with 45 kg hm^-2^ of pure N input. All of the data are means of four replicates.

To illustrate N allocation in the architecture of plants at anthesis and maturity, the percentages of N accumulated in each organ (leaf blades, sheath, stem, and spike) were showed in **Figures 1**, **2**. From anthesis to maturity, the spike was the sink to store N (∼30% at anthesis under HN, ∼50% at anthesis under LN, and ∼80% at maturity under both high and low N), and other organs served as the sources to transport N to the sink. Under HN, there was a ∼50% N increase in the sink of the spike, which came from the source organs in the following order: stem (∼17%), sheath (∼12%), second leaf (∼8%), flag leaf (∼5%), third leaf (∼5%), and other leaves (∼2%). Under LN, there was a ∼40% N increase in the sink of the spike, which came from the source organs in the following order: stem (∼17%), sheath (∼11%), second leaf (∼4%), flag leaf (∼2%), third leaf (∼2%), and other leaves (∼0.7%). When the N supply changed from HN to LN, the stem served as the main source organ of N transportation and maintained very similar percentages of N in the whole plant allocation at both anthesis and maturity. This implies that the stem is the most important organ in plant architecture from anthesis to maturity. The percentages of N in the flag leaf, second leaf, and third leaf decreased in terms of whole-plant allocation at both anthesis and maturity. It is likely that these source organs are heavily affected by a limited N supply. To analyze differences in the responses of organs under HN and LN, the N percentage of each organ was compared among the five mutant lines and the WT at anthesis and maturity. As can be seen in **Figure 1**, there was a sharp decrease (of almost 50%) in the third leaf (significant difference only presented for A1-28, A1-84, and A16-11 when comparing to the WT) and other leaves (significant difference only presented for A1-28, A9-29, and A16-11 when comparing to the WT) at anthesis under LN. Meanwhile, there was a slight increase in the N percentage in the spike of the mutants (significant difference only presented for A1-226 when comparing to the WT) at anthesis under LN. This suggests that the N percentage in leaves below the second leaf was reduced as a result of transfer to spikes, to adapt to N stress. In addition, there were small decreases in N allocation to the sheath and the third leaf at both anthesis and maturity under LN in A1-28 and A16-11 (**Figures 1**, **2**). These two lines showed significant N decreases in the sheath and third leaf at anthesis compared to the WT, while only A1-28 showed a significant decrease in the sheath and third leaf at maturity compared to the WT.

### NUE Characterization of the Five Mutant Lines

The NUE values of the five mutant lines were assessed and are shown in **Table 4**. Four of the five lines (A1-84, A1-226, A9-29, and A16-11) had significant NUE improvement, with increases of 16–41% compared to the WT under HN, and two of the five lines (A1-84 and A9-29) had significant NUE improvement, with increases of 17–25% under LN. In assessing the NUE of each mutant line, two components (NUpE and NUtE) were used to define N uptake and utilization ability separately. The NUpE values of all of the mutant lines with improved NUE compared to the WT (mentioned above) were significantly higher than that of the WT; NUtE values did not improve significantly in any of them, neither with HN nor LN. By contrast, A1-28 had a notable decrease under HN, and A1-84 had a remarkable drop under LN, indicating that NUtE values did not improve in the five mutant lines. The NUtE value was significantly lower in A1-84 than in the WT and the other four lines under LN, whereas the NUpE value was significantly higher than in all of them. These findings indicate that A1-84 is a typical mutant with a strong ability to take up N. Two lines (A1-226 and A16-11) had improved NUE values only under HN, indicating that enhancement of N uptake in these mutants was dependent on the N level supplied. The other two lines (A1-84 and A9-29) had higher NUE values under both HN and LN, indicating that the enhanced N uptake of the two mutants was independent of the level of N supplied.

**Table 4:** Comparison of NUE, NUpE and NUtE between Hua-30 and mutant lines (2014–2015).

Nitrogen regime	Line	NUE (g/g)	NUpE (g/g)	NUtE (g/g)
HN	Hua-30	22.12d	0.40c	55.24a
	A1-28	20.80d	0.48bc	43.34b
	A1-84	25.61c	0.55ab	47.88ab
	A1-226	31.09a	0.63a	49.44ab
	A9-29	29.24ab	0.62a	47.44ab
	A16-11	27.20bc	0.57a	47.93ab
LN	Hua-30	37.77c	0.56c	67.89a
	A1-28	41.22bc	0.66bc	62.99a
	A1-84	44.28ab	1.20a	33.88b
	A1-226	38.22c	0.55c	69.94a
	A9-29	47.36a	0.76b	63.17a
	A16-11	41.43bc	0.62bc	67.61a

*NUE, nitrogen use efficiency; NUpE, nitrogen uptake efficiency; NUtE, nitrogen utilization efficiency. HN indicates the treatment with 160 kg hm^-*2*^ of pure N input, and LN indicates the treatment with 45 kg hm^-*2*^ of pure N input. Different letters within the same column indicate significant differences at *P* < 0.05 by least significant difference (LSD) test. All data are means of four replicates*.

### Translocation of Dry Matter and N in the Five Mutant Lines

To assess the dry matter and N translocation of the five mutant lines from anthesis to maturity, DMT, NT, DMTE, and NTE values were analyzed separately and are listed in **Table 5**. Under HN, there were no significant increases in DMT, NT, DMTE, or NTE in any of the mutant lines compared to the WT. Conversely, the NTE values of A1-28, A1-226, and A9-29 were significantly lower than those of the WT, with decreases of 12–17%. Under LN, there was a significant increase in the DMT and DMTE values of three lines (A1-84, A9-29, and A16-11) compared to the WT, with increases of 90–142% and 62–75%, respectively. In addition, A1-84 had a significantly higher NT value with an increase of 204% and A1-28 had a significantly higher NTE value with an increase of 12% compared to the WT. However, the other lines did not show significant increases in NT or NTE values compared to the WT.

**Table 5:** Comparison of dry matter and nitrogen translocation in Hua-30 and mutant lines.

Nitrogen regime	Line	DMT(g)	DMTE (%)	NT(mg)	NTE (%)
HN	Hua-30	6.06a	30.85a	159.18a	71.37a
	A1–28	6.01a	29.12a	134.22a	62.82bc
	A1–84	5.79a	22.50a	167.48a	68.67ab
	A1-226	4.08a	18.02a	172.89a	61.71bc
	A9-29	5.96a	27.22a	172.79a	59.32c
	A16-11	6.95a	27.57a	178.52a	65.46abc
LN	Hua-30	2.40c	23.86b	36.84b	60.27b
	A1-28	3.83bc	32.85ab	48.51b	67.71a
	A1-84	5.80a	41.65a	112.03a	64.84ab
	A1-226	3.63bc	35.04ab	42.78b	62.98ab
	A9-29	5.40ab	39.19a	50.34b	61.11b
	A16-11	4.57ab	38.71a	40.38b	61.78ab

*DMT, dry matter translocation; DMTE, dry matter translocation efficiency; NT, nitrogen translocation; NTE, nitrogen translocation efficiency. HN indicates the treatment with 160 kg hm^-*2*^ of pure N input, and LN indicates the treatment with 45 kg hm^-*2*^ of pure N input. Different letters within the same column indicate significant differences at *P* < 0.05 by least significant difference (LSD) test. All data were means of four replicate*.

## Discussion

### Introduction of Mutation and Stabilization of Homozygosity by Microspore Mutagenesis in an Efficient Way

The microspore is a haploid gamete that contains a single copy of each chromosome from diploids. It is totipotent and can divide to form a multicellular structure *in vitro* and then regenerate a fertile plant (Eudes et al., 2014). Therefore, the microspore is an ideal target for mutagenic treatment, which can rapidly generate homozygous mutants (Szarejko and Forster, 2007). In addition, microspores without the anther wall layer that are directly in contact with the mutagen can lead to a high mutation percentage. An early successful case reported in oilseed rape Canola produced five DH mutant lines with tolerance to the herbicide imidazolinone by microspore mutagenesis using ethyl nitrosourea (Swanson et al., 1989). In another successful reported case, in *Brassica carinata*, nine mutant lines with changes in erucic acid concentration were obtained from nearly 400 DH lines produced by microspore mutagenesis using sodium azide (Barro et al., 2001). In barley, 61 mutant lines with morphological and developmental changes were obtained from 564 DH lines produced by microspore mutagenesis using sodium azide (Castillo et al., 2001). Recently, a study in Chinese cabbage produced six stable inheriting mutants from 1304 regenerated plants by microspore mutagenesis using EMS (Huang et al., 2016). In addition to chemical mutagenesis, microspore mutagenesis using radiation has also been successful in *B. napus* (Beaith et al., 2005) and Chinese cabbage (Huang et al., 2014).

In our study, three mutant lines (A1-84, A1-226, and A9-29) with improved NUE under HN were obtained from 329 DH lines produced by microspore mutagenesis using EMS, and one mutant line (A16-11) with improved NUE under HN was obtained from 27 DH lines produced by microspore mutagenesis using PYM, as well as two mutant lines (A1-84 and A9-29) with improved NUE under LN. The rate of generation of mutant lines with high NUE is encouraging. The integration of mutation and mutant stabilization in one step by microspore mutagenesis provides a powerful tool to produce mutant lines that are ready for direct screening. Limited-N treatment in microspore embryogenesis seemed to improve the screening of high NUE plants in our experiment, the mechanism of which is worthy of further investigations.

### Characterization of Mutant Lines With Multiple Parameters Related to NUE

NUE is a complex genetic trait implicated in N uptake, translocation, assimilation, and remobilization (Xu et al., 2012). According to Moll et al. (1982), NUE can be divided into NUpE and NUtE. It is difficult to describe or define a mutant with improved NUE based on the visual characteristics of the crop in field experiments. However, NUE under different regimes can be analyzed. For barley and other cereal crops, the grain-filling stage is very important for the establishment of yield. Therefore, the periods of anthesis and maturity were chosen for the NUE analysis in this study. The parameters related to NUE, including NUpE, NUtE, and NTE, are useful to characterize mutants in terms of N uptake, utilization, and transportation. After analyzing five mutant lines using these parameters, no line showed improved NUtE, but four lines (A1-84, A1-226, A9-29, and A16-11) showed enhanced N accumulation under HN, and two lines (A1-84 and A9-29) showed a greater ability to accumulate N under LN. Meanwhile, our data show that A1-84 had enhanced N accumulation due to having the highest NUpE and lowest NUtE values. Although A1-28 was not outstanding in assessment, it showed remarkable NTE under LN conditions. The difference of those physiological characters between mutant lines under different N levels implies that there are some changes on the limiting factors in plant metabolism related to NUE. It awaits further investigation on the genetic control.

### Strategy for Field Screening of NUE Mutants in Crops

An effective screening strategy was developed and optimized to determine which mutant lines had improved NUE. In the first round of field screening of 356 completely homogenous DH lines, the PTN of single plants was determined as an index for further screening, based on its high positive correlation with the grain yield of single plants under both HN and LN. In the second round of field screening, 16 mutant lines were selected based on their high PTN values over four consecutive years under LN conditions. Finally, five mutant lines were comprehensively assessed to elucidate the characteristics related to NUE. In this study, we quantified dry matter and N in plant tissues to analyze NUE. When comparing the accumulation of dry matter and N among five mutant lines and the WT, the differences showed a similar trend in most cases (for either HN or LN). Both dry matter and N accumulation improved significantly under HN in four of the five lines (not A1-28) and both dry matter and N accumulation improved significantly under LN in four lines (not A1-226), which implies that accumulation of dry matter may serve as a primary index of N use in barley.

N remobilization during the reproductive stage is an important process that determines grain yield (Hirel et al., 2007; Chen et al., 2016). A few studies have shown that it is more effective to select genotypes under LN than HN (Presterl et al., 2003; Hirel et al., 2007). Therefore, it was more important to assess N remobilization from anthesis to maturity under LN. In our study, N remobilization parameters such as DMT, DMTE, NT, and NTE showed greater variation under LN than under HN compared to the WT. This confirmed that LN treatment is a more reasonable selective pressure than HN treatment for barley NUE. A1-84 had the highest accumulation of N in spikes (more than twofold that of the WT) under LN at both anthesis and maturity, which indicates that the quantity of N transferred from vegetative organs (source) to the spike (sink) was higher than in other lines at the filling stage. The reason that the NUtE values of A1-84 under LN were significantly lower than those of the WT can be attributed to the large amounts of N accumulated in shoots (nearly threefold that of the WT) at anthesis. Although the NUtE value of A1-84 was very low (33.88 g/g) under LN, the amount of N in the spike at maturity was still the highest of all lines. It also explained that the grain yields per plant of A1-84 and A1-226 were significantly higher than those of the WT under HN, and only A1-84 showed a significant increase under LN.

### Genetic Improvement of NUtE Required in Future

In this study, four of the five mutant lines (A1-84, A1-226, A9-29, and A16-11) showed significant NUpE improvement under HN and two mutant lines (A1-84 and A9-29) showed significant NUpE improvement under LN, compared to the WT. However, none of the five mutant lines showed any significant improvement in NUtE compared to the WT. It appears that the improvement of NUE relies on better N capture more than N conversion based on the review of UK winter wheat over last 30 years (Sylvester-Bradley and Kindred, 2009), which was consistent with our results. The assessment of NUE for five mutant lines in barley suggested that the improvement of NUE depends on enhancement of N uptake.

Beatty et al. (2010) reported that NUtE plays a predominant role in barley high NUE genotypes under LN, while under HN, NUpE and NUtE played mixed roles in the high NUE genotypes. Similar results have also been obtained in maize (Moll et al., 1982; Moose and Below, 2009). In this study, the role of NUpE was predominant in the mutant lines with improved NUE under both LN and HN. We did not find higher NUtE lines in the mutants under HN. One possible reason is that we did not set up more N treatment levels and another reason may be due to the mutant background used in this study. It would be worth studying more barley genotypes to examine potential high NUtE screening. We anticipate microspore mutation to be an applicable and effective method for screening high NUE plants in other crops.

## Author Contributions

CL, XF, JH, and RL conceived and designed the experiment. RL and GG conducted the microspore culture and generation-adding. RG, CF, and SH performed the agronomic trait investigation, biomass and nitrogen content measurement.RG and JC analyzed the data. RG and CL wrote the manuscript. All authors helped with drafting the manuscript and approved the final manuscript.

## Conflict of Interest Statement

The authors declare that the research was conducted in the absence of any commercial or financial relationships that could be construed as a potential conflict of interest.

## Abbreviations

2, 4-D, 2: 4-dichlorophenoxyacetic acid
6-BA: 6-benzyladenine
CV: coefficient of variation
DH: double haploid
DMA: dry matter at anthesis
DMM: dry matter at maturity
DMT: dry matter translocation
DMTE: dry matter translocation efficiency
EMS: Ethyl methane sulfonate
HN: high nitrogen
KT: kinetin
LN: low nitrogen, LSD, least significant difference
N: nitrogen
NAA: naphthaleneacetic acid
NT: nitrogen translocation
NTE: nitrogen translocation efficiency
NUE: nitrogen use efficiency
NUpE: nitrogen uptake efficiency
NUtE: nitrogen utilization efficiency
PTNs: productive tiller numbers
PYM: pingyangmycin
SDMA: spike dry matter at anthesis
SDMM: spike dry matter at maturity
SNAA: spike nitrogen accumulation at anthesis
SNAM: spike nitrogen accumulation at maturity
TNAA: total nitrogen accumulation at anthesis
TNAM: total nitrogen accumulation at maturity
WT: wild type.
