# Supplementary material for: Rapid Generation of Barley Mutant Lines With High Nitrogen Uptake Efficiency by Microspore Mutagenesis and Field Screening
Source: Front Plant Sci. 2018 Apr 6;9:450. doi: 10.3389/fpls.2018.00450 (PMC5897737; doi:10.3389/fpls.2018.00450)
Supplement: Supplementary file 1 [file Presentation_1.PDF]

frontiers

Supplementary Material

Rapid generation of barley mutant lines with high nitrogen uptake efficiency by microspore mutagenesis and field screening

Runhong Gao, Guimei Guo, Chunyan Fang, Saihua Huang, Jianmin Chen, Ruiju Lu, Jianhua Huang, Xiaorong Fan* and Chenghong Liu*

*** Correspondence:** Chenghong Liu, E-mail: liuchenghong@saas.sh.cn; Xiaorong Fan, E-mail: xiaorongfan@njau.edu.cn

1 Supplementary Figures and Tables

1.1 Supplementary Figures

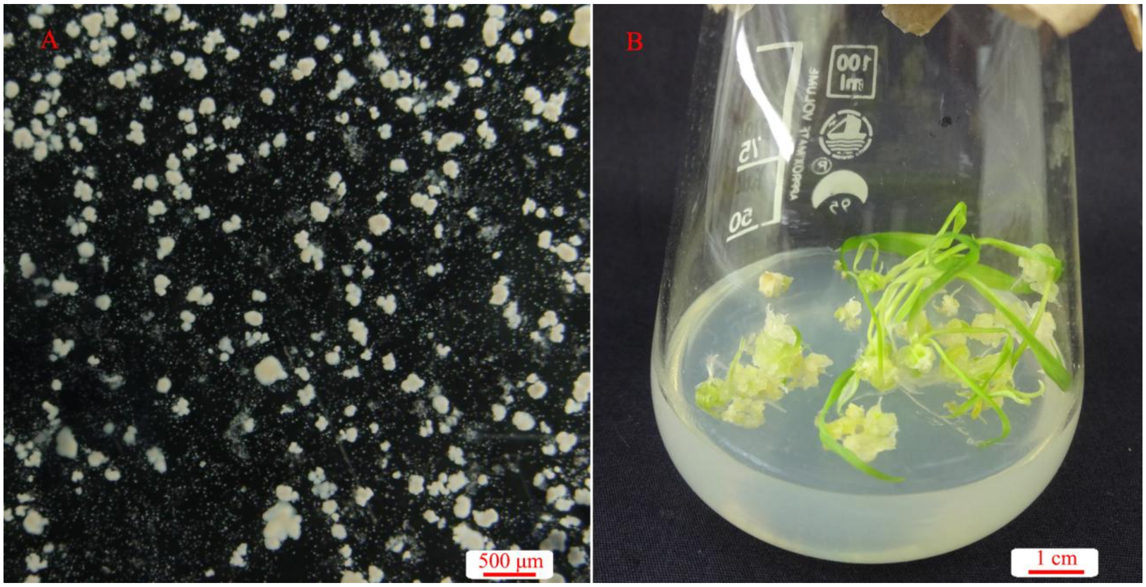

Supplementary Figure 1. Embryogenic callus formation (A, observed by stereomicroscope) from the isolated microspores from barley cv. Hua-30 after 21 d culture and green plant (B) regeneration on differentiation medium after 21 d of culture.

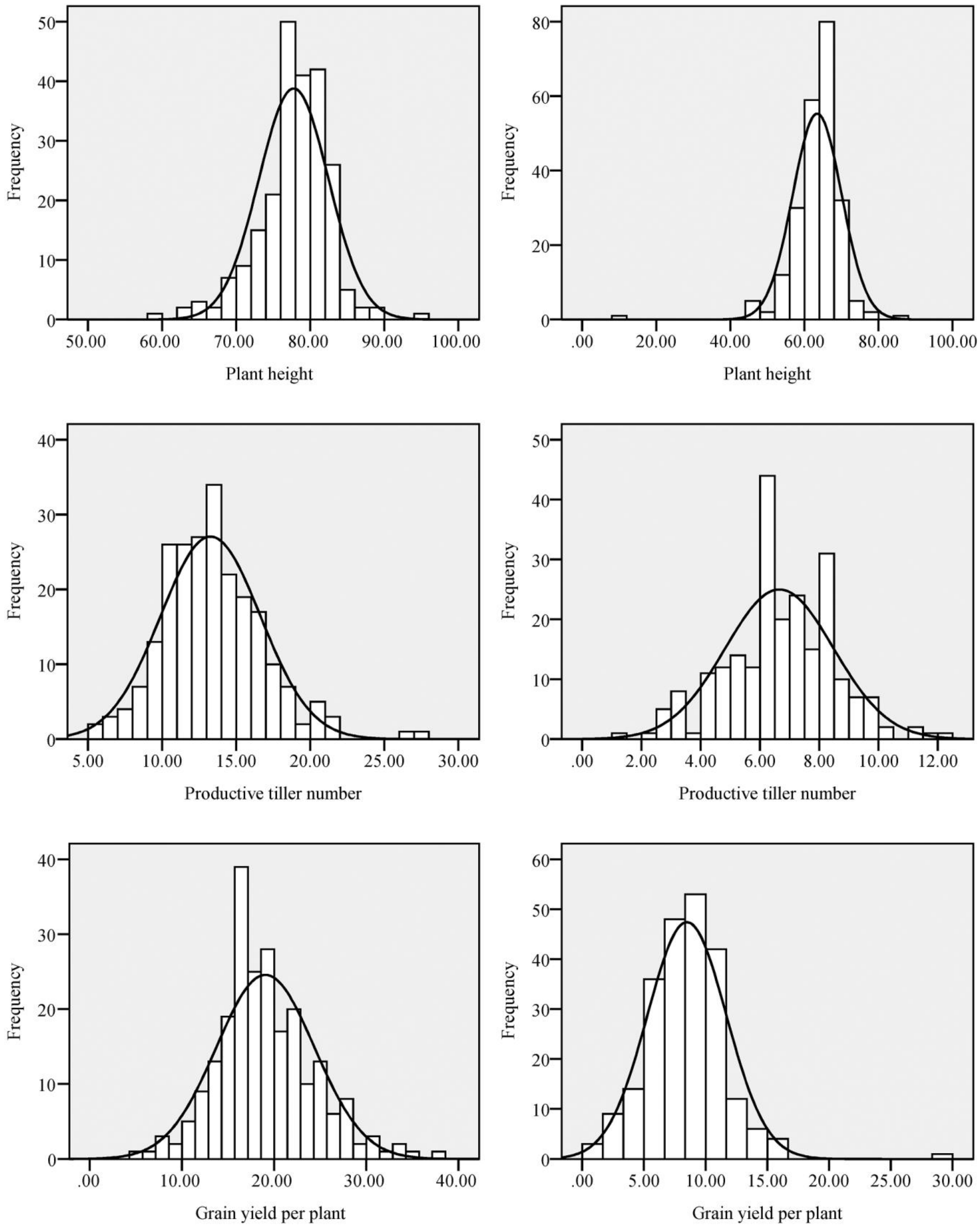

Supplementary Figure 2. The distribution of three agronomic traits under nitrogen-fertilized and nitrogen-unfertilized conditions in the field experiment of 2011–2012, n = 229. Under fertilized conditions, the skew and kurtosis of plant height, productive number and grain yield per plant were (-0.64, 2.13), (0.70, 1.7), and (0.55, 0.88). Without fertilizer, the skew and kurtosis of plant height,

productive number, and grain yield per plant were (-2.58, 18.94), (-0.15, 0.41), and (1.05, 6.69). The three agronomic traits did not comply with the normal distribution (skewness = 0, kurtosis = 0). Nitrogen-fertilized indicates the treatment with 160 kg $\cdot hm^{-2}$ of pure N input, and nitrogen-unfertilized indicates the treatment without N fertilizers input. The figure and results were obtained by IBM SPSS Statistics 21.

1.2 Supplementary Tables

Supplementary Table 1. Three agronomic traits of 229 mutagenized lines derived from barley cv. Hua-30 in the field experiment of 2011–2012

Agronomic traits		N-fertilized	N-unfertilized
Plant height(cm)	Mean	77.8	63.5
	Range	59.2~95.8	35.5~86.6
	CV (%)	6.06	9.40
Productive tiller number	Mean	13.3	6.6
	Range	5.4~27.4	1.0~12.2
	CV (%)	25.44	27.87
Grain yield per plant(g)	Mean	19.1	8.5
	Range	5.4~38.6	0.2~29.0
	CV (%)	27.89	38.25

N-fertilized indicates the treatment with 160 kg·hm^{-2} of pure N input, and N-unfertilized indicates the treatment without N fertilizers input. CV: coefficient of variation = standard deviation / mean, n=229.

Supplementary Table 2. The productive tiller numbers of 16 mutagenized lines and Hua-30 over 4 years

Lines	2011–2012		2012–2013		2013–2014		2014–2015	
	N-fertilized	N-unfertilized	N-fertilized	N-unfertilized	HN	LN	HN	LN
A1–22	13.6±2.3	5.8±1.0	9.8±1.1	5.0±0.5	-	4.2±0.4**	13.3±0.9	7.1±0.5
A1–24	9.2±1.1	6.2±0.5*	9.6±1.7	3.8±0.4*	-	4.3±0.4**	12.4±1.6	6.6±0.6
A1–28	8.5±0.9	7.4±0.7*	11.6±0.9	5.0±0.5	-	4.1±0.4*	12.9±0.8	8.3±0.5*
A1–45	17.6±2.4	7.4±1.7	7.8±1.2*	5.4±0.8	-	4.0±0.7*	13.0±1.6	7.7±0.5
A1–56	16.0±2.5**	7.4±0.7*	7.80±0.7	4.0±0.8	-	4.0±0.5*	12.9±1.5	6.5±0.1
A1–84	12.8±4.3	9.0±1.6	10.0±1.0	7.2±1.2	12.1±1.3**	4.5±0.5**	13.6±1.2	8.2±0.7*
A1–86	10.8±2.1	6.4±1.3	9.0±0.6	4.2±0.2*	-	4.0±0.4*	12.8±0.9	6.0±0.1*
A1–99	10.8±1.1	10.2±0.6**	11.4±1.5	4.0±0.0*	-	4.4±0.6**	11.9±1.0	6.6±0.5
A1–104	12.0±2.7	8.2±1.2*	10.8±1.1	4.6±0.9	-	3.9±0.5*	13.2±0.7	6.3±0.4
A1–108	12.4±4.1	6.6±1.1	10.4±0.6	4.8±0.4	-	4.0±0.3*	13.2±1.4	6.5±0.3
A1–125	11.2±3.5	5.6±0.8	10.4±1.3	5.0±0.3		5.7±0.5**	15.6±1.2*	6.5±0.9
A1–226	-	-	16.4±1.8**	8.8±0.7**	8.2±0.7	3.6±0.2*	13.0±1.7	6.0±0.3
A9–29	13.8±1.7	9.6±2.0*	10.6±1.9	4.6±0.7		3.3±0.4	12.2±0.9	6.8±0.6
A10–2	14.2±3.0	5.4±0.9	13.2±1.5	4.6±0.7	-	3.9±0.6*	13.6±0.8*	6.7±0.2
A10–11	17.2±2.7**	6.0±0.8	11.0±0.8	6.2±1.0	-	3.9±0.4*	13.8±1.2	6.5±0.2
A16–11	10.6±0.9	8.2±1.0*	14.2±1.3*	9.0±1.9*	8.5±1.1	4.5±0.4*	16.5±1.3*	8.5±0.9
Hua-30	9.8±1.9	4.8±0.6	10.4±0.9	5.2±0.4	8.4±0.7	2.96±0.13	12.4±0.5	6.8±0.3

N-fertilized indicates the treatment with 160 kg·hm^{-2} of pure N input, and N-unfertilized indicates the treatment without N fertilizers input. HN indicates the treatment with 160 kg·hm^{-2} of pure N input, and LN indicates the treatment with 45 kg·hm^{-2} of pure N input. Data are presented as means ± standard deviation (n≥5 in 2011–2013 and n≥15 in 2013–2015); –indicates data deficient; * and ** indicate significant differences at P<0.05 and P<0.01, respectively, compared to Hua-30 by the *t*-test.
